# Function of obturator prosthesis after maxillectomy and prosthetic obturator rehabilitation^☆^^☆☆^^☆☆☆^

**DOI:** 10.1016/j.bjorl.2015.10.006

**Published:** 2015-11-06

**Authors:** Cheng Chen, Wenhao Ren, Ling Gao, Zheng Cheng, Linmei Zhang, Shaoming Li, Pro Ke-qian Zhi

**Affiliations:** a Department of Oral Maxillofacial Surgery, Stomatology Hospital of Xi’an Jiaotong University College of Medicine, Xi’an, Shaanxi, China; b Key Laboratory of Environment and Genes Related to Diseases, College of Medicine, Xi’an Jiaotong University, Xi’an, Shaanxi, China; c Department of General Dentistry, Stomatology Hospital of Xi’an Jiaotong University College of Medicine, Xi’an, Shaanxi, China; d Department of Oral Maxillofacial Surgery, Stomatology Hospital of Xi’an Jiaotong University College of Medicine, Xincheng District, China

**Keywords:** Mouth neoplasms, Maxillofacial prosthesis, Recovery of function, Questionnaires, Neoplasias da boca, Prótese maxilofacial, Recuperação da função, Questionários

## Abstract

**Introduction:**

Maxillary defects are usually rehabilitated by a prosthetic obturator.

**Objective:**

This study aimed to evaluate the functioning of obturators prosthesis in patients with unilateral defects after maxillectomy.

**Methods:**

Of 49 patients, 28 underwent to maxillectomy as a result of tumor ablative surgery, and acquired unilateral maxillary defects. Evaluation of the function was performed by applying the Obturator Functional Scale (OFS).

**Results:**

From a total of 49 patients, 28 were treated as follows: 9 with a conventional retained obturator prosthesis (COP), 11 (39%) with an enhanced retentive obturator prosthesis with stud attachment (POP) and 8 (28%) with an enhanced retentive obturator prosthesis with magnetic attachment (POM). The mean OFS score was 80. Scores on functions of speech, swallowing and chewing reached statistical significances (*p* < 0.05) among these three subgroups. Comparing COP and MOP groups, the scores of OFS in the domains of “Speech-ability to speak in public” and “Swallowing-leakage with liquids” were significantly higher in AOP group. Comparing COP group, the scores of OFS in “Swallowing-leakage with solid” and “Chewing/eating” domains were increased significantly (*p* < 0.05) both in MOP and AOP groups.

**Conclusion:**

Obturator prosthesis improves oral function of patients after maxillary defects; the retention of the obturator prosthesis enhanced by the addition of attachments showed more benefits in oral function.

## Resumo

**Introdução:**

Em geral, os defeitos da maxila são reabilitados por uma prótese obturadora.

**Objetivo:**

O estudo avaliou o funcionamento da prótese obturadora em pacientes com defeitos unilaterais após maxilectomia.

**Método:**

De 49 pacientes, 28 foram submetidos a maxilectomia como cirurgia de ablação tumoral e tiveram como sequela defeitos maxilares unilaterais. A avaliação do funcionamento foi efetuada pela aplicação da Escala Funcional do Obturador (EFO).

**Resultados:**

De um total de 49 pacientes, 28 foram tratados da seguinte forma: nove com prótese obturadora retentiva convencional (POC), 11 (39%) com prótese obturadora retentiva com fixação por pino (POP) e oito (28%) com prótese obturadora retentiva com fixação magnética (POM). O escore médio na EFO foi de 80. Os escores para funções da fala, deglutição e mastigação alcançaram significância estatística (p < 0,05) entre os três subgrupos. Na comparação entre os grupos POC e POM, os escores da EFO nos domínios da “Fala-capacidade de discursar em público” e “Deglutição-vazamento de líquidos” foram significativamente mais altos no grupo POP. Na comparação com o grupo POC, os escores da EFO nos domínios de “Deglutição-vazamento com sólido” e “Mastigação/ingestão” estavam significativamente aumentados (p < 0,05) nos grupos POM e POP.

**Conclusão:**

A prótese obturadora melhorou o funcionamento oral de pacientes com defeitos maxilares; a retenção da prótese obturadora reforçada pela adição de dispositivos de fixação demonstrou maiores benefícios na função oral.

## Introduction

The survival rates of patients with head and neck cancer have improved in past several decades.^1^, ^2^, ^3^ Functional rehabilitation and quality of life (QOL) after maxillofacial surgery have been emphasized in recent years. It mainly depends on outcomes of maxillofacial reconstruction and rehabilitation involving functions, esthetics, psychology acceptance and resocialization.^4^ Prosthetic obturator was the primary method employed in rehabilitating larger maxillary defects.^5^, ^6^, ^7^, ^8^ The aim was to close the defect, separate the oral cavity from nasal cavities and prevent hyper-nasal speech, nasal regurgitation of food and liquids, and support the facial profile. Based on location and size of defect, health conditions of remaining teeth and bones, available soft tissue undercuts and muscular control, various obturator prostheses with different retentive designs were used to improve oral functions.

Besides conventional retained obturator prosthesis, others with various retentive types^9^, ^10^, ^11^ for enhancing stability and retention of prosthesis have been widely used. It was reported by some researchers that obturator prosthesis with enhanced retention can improved oral functions postoperatively. But compared to conventional prosthesis, controversy exists whether the reinforced design of obturator had more benefits.^12^ There were few studies in comparison and evaluation of various obturator prostheses functionally after maxillary defects.^13^

In this study patients with similar unilateral maxillary defects were treated with three types of obturator prostheses. Obturator Functional Scale (OFS) questionnaires was applied to assess oral functions among the three subgroups.

## Methods

This historical cohort study was conducted at Department of Head and Neck Cancer of Xi’an Jiaotong University Stomatology Hospital in China. Patients, who were treated with maxillectomy for oral cancer which had invaded the maxilla from January 2000 to January 2010, were included in the study. The inclusion criteria were: maxillectomy patients and patients who acquired similar unilateral maxillary defects and received rehabilitation of obturator prosthesis. Patients were excluded if they had local recurrence, no prosthetic rehabilitation, and cognitive impairment or if they had difficulty in physical condition or availability for an extra visit. One year after rehabilitation with obturator prosthesis the patients were asked to complete the OFS for function evaluation. Forty-nine patients met the criteria. The study was approved by the local ethical board of the College of Medicine (n° XJTULAC2014-203), Xi’an Jiaotong University and informed consent was obtained. Based on medical records, the condition of preoperative dentition was considered good when no more than 6 maxillary teeth were missing before surgery and was considered poor when 6 or more maxillary teeth were missing before surgery.^14^ Sizes of maxillectomy defects were established from the operative and radiographic records using the Brown classification.^15^ Based on retentive types, All patients in this study were divided into 3 subgroups: patients who received conventional retained obturator prosthesis (COP), patients who received enhanced retentive obturator prosthesis with stud attachment (AOP) and patients who received enhanced retentive obturator prosthesis with magnetic attachment (MOP).

### Obturator function scale

The OFS was used to assess the self-reported function of obturator prostheses. Eight domains in OFS questionnaires, including satisfaction with facial appearance, ability to speak in public, leakage with liquids and solids, dryness of mouth, insertion of an obturator, chewing or eating, social–family interactions and overall OFS, were scored. Responses to OFS were completed by telephone or subsequent visits. Numerical value from 0 to 100 for each response in the questionnaires was used. A score of 0 indicates maximum suffering or dissatisfaction and a score of 100 indicates that the patient was asymptomatic or extremely satisfied in the respective domain.^16^ The questionnaires have been validated and used by other investigators.^17^, ^18^ Internal consistency of the questions was assessed by Cronbach's alpha test. The English version of OFS questionnaires were created by Chigurupati and translated into Chinese version for investigation. The Chinese version of OFS was also validated in China via reliability test and used in other research.^19^

### Statistical analysis

The data of medical records was listed in Table 1. Age, sex, tumor diameter, pathology diagnosis, chemical therapy, radiation therapy, premorbid dentition and model of enhanced retention were selected as demographic and treatment variables. Data gathered from patient responses to OFS were scored and analyzed. The impact of select demographic and treatment variables on OFS was assessed: 1 – age (>60 or ≤60 years); 2 – sex (male or female); 3 – chemotherapy (yes or no); 4 – postoperative radiation therapy (yes or no); 5 – premorbid dentition (good or poor). Statistical analysis was performed using nonparametric Kruskal–Wallis rank sums analysis and post hoc analysis with SPSS 18.0. Correlation analysis between OFS and demographic variables was calculated by likelihood ratio in ANOVA analysis with SPSS 18.0. The results were considered significant at *p* < 0.05.

**Table 1:** Social and medical characteristics of patients.

Variables	*n* = 28
Age (*X* ± SD)	62.05 ± 8.84 (47–81)
Sex (M/F)	19/9
Tumor diameter (*X* ± SD, mm)	4.2 ± 1.4 mm
Pathology diagnosis	5/23
Saliva adenoid cystic carcinoma	11
Squamous cell carcinoma	7
Mucoepidermoid carcinoma	5
Myoepithelial adenoma	2
Pleomorphic adenoma	1
Ameloblastoma	2
Chemical therapy	10
Radiation therapy	2
Premorbid dentition (G/P)	18/10
Brown classification (2a/2b)	15/13
Model of enhanced retention	
Conventional designed	9
Attachment enhanced	11
Magnet enhanced	8

F, female; M, male; B, benign; M, malignance; G, good; O, poor; X, sample mean; SD, standard deviation.

## Results

Of the 49 patients, 4 patients had died, and 6 patients who did not contact by telephone or postal mail were excluded. Of the 39 patients, 28 patients were included; 6 patients with acquired bilateral or small defects were eliminated; 5 patients had other problems (advanced age and poor physical condition). The age of the patients ranged from 47 to 81 years (mean, 62.05 years; standard deviation [SD] 8.84 years). Time elapsed from maxillectomy and rehabilitation of prostheses to OFS response in this study ranged from 1.8 to 6.8 years (mean, 2.5 years; SD, 1.3 years). Sixty-eight percent of the patients were men, and the mean age was 61 years. Of the 28 patients, 11 patients were diagnosed as salivary adenoid cystic carcinoma (SACC); 7 patients were diagnosed with squamous cell carcinoma (SCC); 5 patients were diagnosed as mucoepidermoid carcinoma; 2 patients were diagnosed as myoepithelial adenoma; 2 patients were diagnosed as ameloblastoma and only 1 patient was diagnosed as pleomorphic adenoma. Thirty-six percent (10 of 28) received postoperative chemotherapy and 2 patients received radiotherapy. The preoperative dentition was good in 64% (18 of 28) of patients. Based on models of enhanced retention, of the 28 patients, 9 patients (32%) received COP, 11 patients (39%) received AOP and 8 patients (28%) received MOP.

The mean scores of each domain in OFS are presented in Table 2. The mean score on OFS was 80 (SD, 14.1). “Speech” and “Insertion” were fairly high, with mean scores of 85.82 (SD, 19.0) and 83.93 (SD, 23.78), respectively. “Swallowing-leakage with solids” and “Chewing/eating” were fairly low, with mean scores of 59.68 (SD, 16.87) and 63.21 (SD, 24.70). Scores on functions of speech, swallowing and chewing reached statistical significances (*p* < 0.05) among three subgroups. Further analysis revealed that different retentive obturator prostheses resulted in different efficiency of rehabilitation. Comparing COP and MOP groups, scores of OFS in the domains of “Speech-ability to speak in public” and “Swallowing-leakage with liquids” were significantly higher in AOP group. While comparing COP group, scores of OFS in domains of “Swallowing-leakage with solid” and “Chewing/eating” were increased significantly (*p* < 0.05) both in MOP and AOP groups. Furthermore, overall OFS score reached statistical significance in AOP group.

**Table 2:** Score of OFS questionnaires in patients.

Domains of OFS	Total	Subgroups			*p*-Value
	(*n* = 28)	COP (*n* = 9)	AOP (*n* = 11)	MOP (*n* = 8)	
Satisfaction with facial appearance	65.18 ± 22.91	69.44 ± 20.83	68.18 ± 22.16	56.25 ± 25.88	0.13
Speech	85.82 ± 19.00	81.56 ± 24.22	91.00 ± 15.41	83.50 ± 17.64	0.05
Speech-ability to speak in public	64.43 ± 23.98	55.78 ± 23.75	78.91 ± 22.45^b^	54.25 ± 17.59^b^	0.02^a^
Swallowing-leakage with liquids	66.82 ± 20.39	59.44 ± 14.99	78.91 ± 22.45^b^	58.50 ± 15.73	0.01^a^
Swallowing-leakage with solids	59.68 ± 16.87	44.33 ± 17.00	70.00 ± 9.94^b^	62.75 ± 12.02^b^	0.01^a^
Chewing/eating	63.21 ± 24.70	40.67 ± 22.44	79.00 ± 16.65^b^	66.88 ± 17.91^b^	0.01^a^
Saliva-dryness of mouth	75.21 ± 17.19	78.00 ± 16.50	69.91 ± 18.00	79.37 ± 17.07	0.07
Insertion of obturator	83.93 ± 23.78	77.78 ± 26.35	81.82 ± 25.22	93.75 ± 17.67	0.02^a^
Social family interaction	60.89 ± 16.08	59.44 ± 14.99	63.81 ± 18.14	58.50 ± 15.74	0.03^a^
Overall OFS score	80.00 ± 14.40	68.89 ± 14.53	89.09 ± 10.44^b^	80.00 ± 10.69	0.01^a^

OFS, Obturator Function Scale; AOP, attachment retained obturator prosthesis; COP, conventional obturator prosthesis; MOP, magnetic obturator prosthesis.

a Statistical significance, *p* < 0.05(Kruskal–Wallis rank sums analysis).

b Statistical significance, *p* < 0.05. A post hoc analysis reached significance among COP group, AOP group and MOP group (Duncan sums analysis).

The impact of the select demographic and treatment variables on OFS domains are presented in Table 3. Overall OFS correlated significantly with postoperative radiotherapy and premorbid dentition (*p* < 0.05). Furthermore, scores on “interaction”, “speech in public” and “satisfaction appearance” correlated with premorbid dentition.

**Table 3:** Impact of treatment and demographic variables on selected individuals.

Predictor variable	Patients n°	Domains	Mean score (SD)	*p*-value
Sex	16 (male)	None^a^		
	13 (female)			
Chemical therapy	18 (no)	None		
	10 (yes)			
Age	9 (<60)	None		
	19 (≥60)			
Brown classification	15 (2a) 13 (2b)	None		
Postoperative radiotherapy	26 (no)	Overall OFS	82.31 ± 11.77	0.01^b^
	2 (yes)		50.00 ± 14.14	
Premorbid dentition	18 (good)	Overall OFS	83.33 ± 14.14	0.02^b^
	10 (poor)		74.00 ± 13.50	
		Interaction	68.83 ± 7.78	0.01^b^
			46.60 ± 17.56	
		Speech in public	63.00 ± 30.12	0.01^b^
			67.00 ± 0.00	
		Satisfaction appearance	66.67 ± 21.00	0.04^b^
			62.50 ± 27.00	

SD, standard deviation; OFS, Obturator Functioning Scale.

a No significant correlations in the all OFS domains.

b *p* < 0.05, significant correlation.

## Discussion

The obturator prostheses were mainly used in rehabilitating maxillary defect.^8^ Theoretically, well-designed obturator prostheses for maxillary defects were not only to maintain durable and good retention, stability, and support, but also to relieve pain and result in ease of use. One of the most crucial parts for application of obturator prosthesis is the retention of prosthesis.^20^ With the development of research and improved techniques, there were various strategies, designs and materials to achieve enhanced retention, such as precise attachment supported by implant retentive obturator prosthesis.^21^, ^22^ In this study, we evaluated retrospect data of medical records over past 10 years in our department. The strongest predictor of OFS was retentive type; OFS correlated strongly with condition of remaining teeth and radiation therapy.

It was evident by previous studies that location and size of the maxillectomy defect tremendously influenced functions of obturator prostheses.^17^, ^23^ Rogers et al.^24^ reported that patients with larger defects had lower scores for activity, recreation, physical function. Okay et al.^25^ concluded that stability of prosthesis was compromised as the defect size increased, resulting in poor obturator function. Brown and Shaw^15^ summarized that obturator reconstruction was offered to patients with Class 1 to 2a and 2b defects, but a composite free flap option was preferred for larger alveolar and Class 3 or 4 defects, when appropriate to the patient's medical fitness and personal choice. But Chigurupati et al.^14^ reported that the size of defect did not correlate with obturator function on those patients who acquired maxillary defects (Brown Class 2a or 2b). Similar with Chigurupati's report, our study showed that oral functions were correlated with size of defects. This might be due to improvement of surgeon's reconstruction plan according to previous treatment experience. Additionally in order to reduce bias, installation and fabrication of obturator prostheses of all patients were finished only by single experienced prosthodontist and single dental technician. The prosthodontist checked oral conditions of patients, studied residual bone and teeth preoperatively, and evaluated conditions of remaining teeth.

Previous investigators have confirmed that the number of remaining abutment teeth and periodontal health played a vital role in stability and retention of prosthesis.^26^ The distance of the direct retainer to the fulcrum line of the prosthesis can also affect the stability of the obturator. Of the 28 patients, 64.3% had good conditions of residual teeth and residual alveolar bone (mesial to defects). In accordance with a previous report,^17^ in this series condition of dentition influenced severely function of obturator. There were significant correlations between dentition and domains of OFS. So we recommended that remaining teeth should be evaluated and treated before operation. Adding extra retentive appliance also requires healthy teeth.

Many studies report that patients, who rehabilitated by obturator prosthesis after maxillectomy, the receipt of postoperative radiation was the principle negative factor on quality of life.^14^ Patients with malignant tumors who received postoperative radiation therapy developed significant trismus, difficulty with obturator insertion, and dryness and soreness of the oral mucosa. The overall OFS in patients with radiation therapy was significantly lower than others. But only 2 patients in this study received radiation therapy, so the influences by radiation therapy lack support.

**Figure 1:**
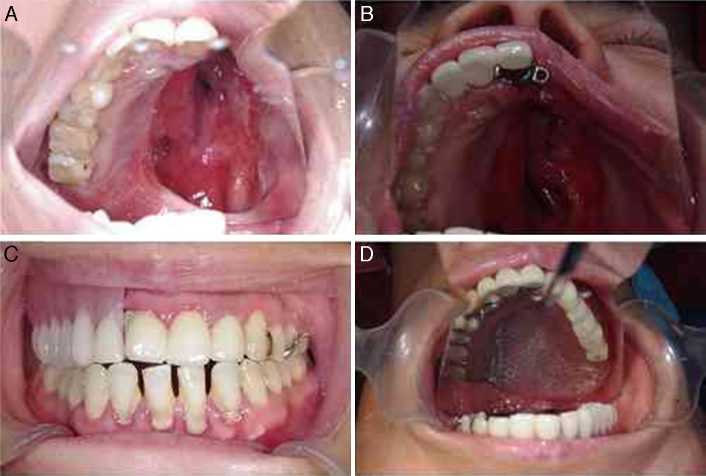
(A) Image before definite rehabilitation; (B) view of try-in of the fixed part; (C and D) view of the definite prosthesis with stud attachment.

OFS was finished within the second year after rehabilitation to analyze the differences. In this study, compared with other groups, the AOP group demonstrated better scores in all domains of OFS, particularly in speech, chewing and swallowing. Patients with obturator prosthesis which was enhanced by stud attachment do better than conventional and magnetic retentive prosthesis in improving oral function, especially in speech and swallowing. Consistent with our reports, the function of obturator was enhanced when adding attachment reported by other investigators.^27^ The retention of conventional prostheses mainly depends on setting various clasps to healthy abutment; and the stability gained from remaining teeth and bone. But clasps have a horizontal force instantaneously to abutment teeth caused by cycles of insertion/removal, and may lead to chronic periodontal damage inevitably.^28^ Thus the tooth adjacent to the defect suffered many overburden outside forces, resulting in rapid periodontal damages of the tooth. For delivering the stresses directed at the primary abutment teeth, fixation of some or all of the remaining teeth may be beneficial. Stud attachments were economical, easy-replaced, and one of the most important benefits was reduction of the unbalanced stress on the abutment teeth. In this study, a porcelain fused metal continuous bridge was based on lowering the burden of the abutment tooth adjacent to defect (Fig. 1). In consideration of the large one-side defect and to the sex of the patient, in female patients stud attachment might be a better choice for enhancing retention and improving esthetics and patient's confidence.

**Figure 2:**
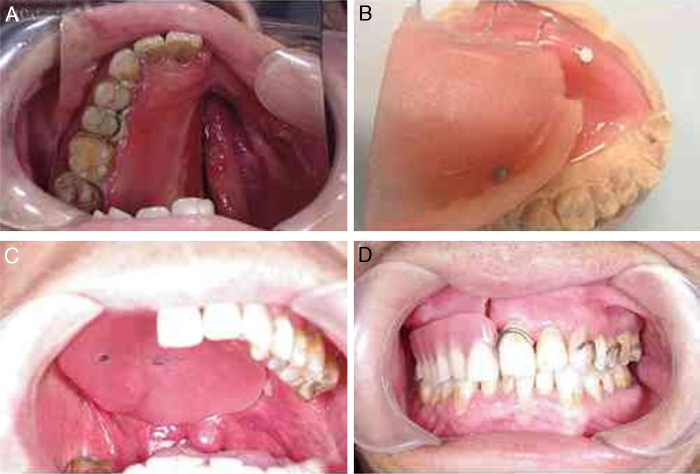
(A) View of defects before definite rehabilitation; (B) image of tissue surface of obturator and magnetic attachment; (C) set-up of obturator; (D) intraoral view of the denture.

Prosthesis retained by magnetic attachment was widely used in strengthening retention. In this series, compared with other groups, OFS scores were higher in “Insertion of obturator” and “Saliva-dryness” in MOP group. It is obviously that separated denture and obturator were easier to set up or remove (Fig. 2). But there was no statistical significance among three groups. The reason might be that the obturator for closure of the nasal defect usually is created larger in volume and weight. Although it could obtain better retention and closure at rest, stability was compromised immediately when exercised functionally after insertion of the under part of prosthesis.

The small sample and duration of follow-up are the main limitations in this study, but it brings new insight related to prosthetic obturation of maxillary defects after surgical resection of malignant tumors of oral cavity, more specifically the hard palate. The lack of long term follow-up and multivariate analysis for other important predictors and treatment variables are the other limitations in this study. A longitudinal, prospective study with a large sample should be considered in the future.

## Conclusion

Obturator prostheses improve oral function of patients after maxilla defects; retention of the obturator prostheses enhanced by the addition of an attachment shows more benefits in oral function.

## Funding

This study was supported in part by the Key Science and Technology Program of Shaanxi Province (n° 2010ZD KG-50), the Project of Technological Innovation Planning in Shannxi Province (n° 2012KTCL03-17), the Specialized Research Fund for the Doctoral Program of Higher Education of China and Natural Science Foundation of China (n° 81272957 and n° 81171398).

## Conflicts of interest

The authors declare no conflicts of interest.
